# Remodeling of Macrophages in White Adipose Tissue under the Conditions of Obesity as well as Lipolysis

**DOI:** 10.1155/2021/9980877

**Published:** 2021-08-30

**Authors:** Xiaohui Tong, Lu Wei, Tongsheng Wang, Rongchun Han

**Affiliations:** ^1^School of Life Sciences, Anhui University of Chinese Medicine, 350 Longzihu Road, Xinzhan District, Hefei 230012, China; ^2^School of Life Sciences, Hainan University, 58 Renmin Avenue, Meilan District, Haikou 570228, China; ^3^School of Pharmacy, Anhui University of Chinese Medicine, 350 Longzihu Road, Xinzhan District, Hefei 230012, China

## Abstract

Adipose tissue macrophages (ATM) are a major source of low-grade inflammation in obesity, and yet reasons driving ATM accumulation in white adipose tissue (WAT) are not fully understood. Emerging evidence suggested that ATM underwent extensive remodeling in obesity. In addition to abundance, ATM in obesity were lipid-laden and metabolically reprogrammed, which in turn was tightly related to their functional alterations and persistence in obesity. Herein, we aimed to discuss that activation of lipid sensing signaling associated with metabolic reprogramming in ATM was indispensible for their migration, retention, or proliferation in obesity. Likewise, lipolysis also induced similar but transient ATM remodeling. Therefore, we assumed that obesity might share overlapping mechanisms with lipolysis in remodeling ATM. Formation of crown-like structures (CLS) in WAT was presumably a common event initiating ATM remodeling, with a spectrum of lipid metabolites released from adipocytes being potential signaling molecules. Moreover, adipose interlerkin-6 (IL-6) exhibited homologous alterations by obesity and lipolysis. Thus, we postulated a positive feedback loop between ATM and adipocytes via IL-6 signaling backing ATM persistence by comparison of ATM remodeling under obesity and lipolysis. An elucidation of ATM persistence could help to provide novel therapeutic targets for obesity-associated inflammation.

## 1. Introduction

The prevalence of Western dietary pattern and sedentary lifestyle boosted the pandemic of obesity worldwide, which was closely associated with increased risks of various metabolic disorders [1]. To tackle this problem, the development of new therapeutics for obesity is urgent. Strongly involved in the pathogenesis of obesity was white adipose tissue (WAT) [2]. Expansion of fat in obesity was associated with adipose tissue macrophages (ATM) accumulation, which caused a state of chronic, low-grade inflammation, and insulin resistance [3]. To date, reasons for the persistence of excessive ATM in obesity have not been fully understood. Canonically, it was known that inflamed adipose tissue could overexpress chemokines such as monocyte chemoattractant protein-1 (MCP-1) which would lead to increased infiltration of macrophages [4]. Despite the role in inflammation, ATM were also key players for systemic energy homeostasis as lipid scavengers. ATM would increase the uptake of lipid fluxes from adipocytes in mice in response to fasting or pharmacologically induced lipolysis, while intra-abdominal ATM depletion resulted in elevated fasting serum free fatty acid (FFA) levels in mice [5]. The fact that most of ATM were localized within crown-like structures (CLS) in obesity [6] indicated CLS might be a vital initiator for obesity-related ATM recruitment. Indeed, adipocyte death per se can trigger ATM infiltration [7] because ATM were important in digesting dead adipocytes within CLS via secretion of cytokines or lysosomal lipases which activated lipolysis and breakdown of triglyceride (TG) in adipocytes [8, 9]. The group of Becker identified a “metabolically activated” (MMe) phenotype of ATM in obese mice, which were beneficial by clearing dead adipocytes through lysosomal exocytosis at the late onset of obesity [10].

Obesity influenced not only ATM abundance but also their metabolic process, indicated by accumulated cellular lipid droplets (LD) in ATM [11, 12]. Mechanistically, ATM in obese mice and humans overexpressed various cell surface receptors to uptake lipids derived from adipocytes in CLS, many of which were indispensable for ATM accumulation in obesity [13, 14]. Thus, in this regard, it was postulated that adipocytes could crosstalk with ATM via secretion of diverse lipid metabolites and then activated lipid sensing signaling in ATM, which could be another key mechanism underlining obesity-linked ATM accumulation.

Compared to obesity, the process of adipocyte lipolysis was also accompanied with CLS formation, lipid liberation in adipocytes, and inflammation in WAT [6, 15, 16]. The accumulation of ATM occurred in response to fat mobilization activated by fasting or catecholamine signaling activation in healthy lean mice, which would be restored later possibly due to cease of FFAs fluxes from adipocytes [5]. Moreover, ATM also became lipid-laden during lipolysis [5, 17, 18]. Therefore, lipolysis might share overlapping mechanisms with obesity to recruit and remodel ATM. Thus, we postulated that comparison of ATM remodeling in these two conditions would lead to discoveries of related mechanisms for excessive ATM in obesity.

Adipose interlerkin-6 (IL-6) appeared to be a potential shared molecule exhibiting similar upregulation by both obesity and adipocyte lipolysis [16, 19, 20]. In obese mice, IL-6/signal transducer and activator of transcription 3 (STAT3) signaling played an important role in liberation of FFAs from adipocytes [21]. Recently, Yu et al. demonstrated that ATM in obesity could also crosstalk with adipocytes through secretion of IL-6 [22]. Therefore, in this review, supported with current literature, we proposed the existence of a positive feedback loop between adipocytes and ATM via IL-6 signaling in obesity and WAT lipolysis concerning ATM persistence, in hoping to provide new insights for studies of obesity-related inflammation.

## 2. Remodeling of ATM in Obesity

### 2.1. Metabolic Reprogramming of ATM by Obesity

**Figure 1:**
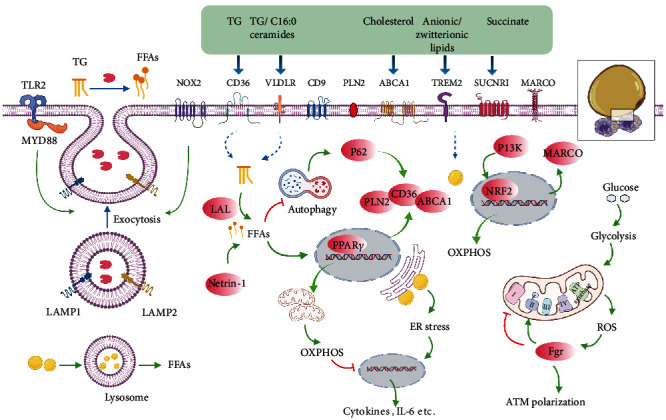
Integrative model of ATM remodeling in obesity. Representation of ATM surrounding apoptotic adipocytes in CLS in obesity, with the small black square enlarging alterations of ATM in connection with dysfunctional adipocytes. The signaling pathways that link these components by activation (green arrows) or inhibition (red inhibitory lines) are also indicated. Briefly, in obesity, ATM are dynamically recruited to apoptotic adipocytes to form CLS and becoming lipid lading associated with increased synthesis of lysosome responsible for catabolism of LD. This alteration might involve these following processes. Firstly, to digest dead adipocytes and facilitate FFA liberations, exocytosis of lysosomal hydrolases from ATM is activated by NOX2 and TLR2/MYD88 signaling pathways with the detection of LAMP1 and LAMP2 on cell surface of ATM. Secondly, expressions of specific cell surface markers including CD36, VLDLR, CD9, PLN2, ABCA1, TEM2, SUCNR1, and MARCO are boosted in ATM in response to stimulation of metabolites like TG, ceramides, cholesterol, anionic/zwitterionic lipids, or succinate, contributing to increased lipid uptake and LD formation in ATM, which seems to be essential for ATM migration and accumulation in adipose tissue by obesity. Mechanistically, metabolism of cellular TG to FFAs mediated by LIPA activates PPAR*γ* responsible for increased expressions of PLN2, CD36, and ABCA1, while activation of PI3K-NRF2 signaling leads to increased MARCO expression. Finally, lipid accumulation in ATM might lead to metabolic reprogramming associated with ER stress as well as shifting to aerobic glycolysis in which building up of ROS results in Fgr activation, mitochondrial complex II activation, and complex I inhibition, all of which induce preferential production of inflammatory cytokines and ATM polarization. By contrast, OXPHOS promoted by FFA metabolism and p62 activation due to reduced autophagy or PI3K signaling activation exhibits anti-inflammatory functions.

The initial event leading to infiltration, retention, or proliferation of ATM under obesity was of limited understanding. The phenomenon that up to 90% of ATM was localized within CLS in adipose tissue in obese *db/db* mice and obese humans [6] implied CLS might have a pivotal role in obesity-associated ATM accumulation. By means of immunofluorescence, Haase et al. demonstrated that the microenvironment of CLS predominantly contributed to a time-dependent increase in ATM proliferation in mice fed with high fat diet (HFD) [23]. At the late state of obesity, expansion of fat was dominated with CLS clustering and adipocyte death in obese mice [10, 24]. Meanwhile, the number of ATM could rise up to 10-folds in adipose tissue in diet-induced obesity (DIO) in mice [3]. Thus, obesity-linked CLS formation was possibly one of the events orchestrating ATM accumulation. Then, what were the signaling molecules within the loci of CLS that gave rise to obesity-associated ATM abundance? It was well acknowledged that obesity was featured by inefficiency of lipid storage and increased lipid liberation from adipocytes in WAT [25], creating a lipid-rich microenvironment where cells including ATM were metabolically challenged. Would this metabolic challenge to ATM relate to their persistence in obesity? (Figure 1).

The concentration of lipids in ATM in obesity was higher than that of the lean counterpart in mice [11, 12, 18]. In fact, obesity-associated ATM were metabolically remodeled to be preferential of lipid handling. Firstly, stimulated by the lipid-rich microenvironment, ATM boosted cellular lipid contents by upregulation of lipid uptake in response to obesity in mice. This process was mediated by increased expressions of a variety of cell surface markers, including the ATP-binding cassette transporters A1 (ABCA1), very low-density lipoprotein receptor (VLDLR), perilipin (PLIN) 2, and cluster of differentiation (CD)36 [13, 14] as well as the scavenger receptor of macrophage receptor with collagenous structure (MARCO) in mouse models of obesity [18]. Moreover, the large quantity of FFAs released from apoptotic adipocytes activated proteins like nicotinamide adenine dinucleotide phosphate (NADPH) oxidase 2 (NOX2), myeloid differentiation primary response 88 (MYD88) and toll-like receptor 2 (TLR2) in ATM, and modeled ATM to increase the uptake of lipids through lysosomal exocytosis indicated by detection of lysosomal associated membrane protein (LAMP)1 and LAMP2 on cell surface of ATM in obese mice [10]. In the process of lysosomal exocytosis, ATM formed close synapse with dead adipocytes and digested them with lysosomal enzymes reported by Haka et al. using mouse models of obesity, human adipose tissue, and ex vivo cell model [26]. In addition, around three percent of ATM in WAT possibly conjugated to form the multinucleated giant cells (MGCs) in order to phagocytize oversized dead adipocytes in CLS in obese patients and mice [27]. Furthermore, biogenesis of lysosome was driven in ATM to enable high rate of lipid catabolism, because inhibition of lysosome function by chloroquine treatment resulted in accumulation of LD in ATM in obese mice [12, 18]. Lipid metabolism in ATM was also altered by obesity. Petkevicius et al. demonstrated that ATM isolated from obese mice and humans exhibited increased rate of de novo phosphatidylcholine (PC) biosynthesis [28]. Consistently, the cholesterol efflux capacity (CEC) was enhanced in ATM in obesity associated with increased expressions of the transporters like ABCA1 compared to that of lean mice [29].

Taken together, the available data suggested that metabolic reprogramming of ATM featuring lipid handling and LD deposition occurred in obesity, which was likely related to CLS formation. We further set out to discuss in detail the implications of metabolic reprogramming in ATM accumulation and inflammation in obesity.

### 2.2. ATM Accumulation Driven by Lipid Sensing Signaling

The finding that the rate of adipocyte death in WAT coincided with ATM accumulation in obese mice suggested a role of ATM in adipose tissue remodeling [8]. Metabolically, the lipid-laden macrophages in insulin-resistant adipose tissue were also regarded as a protective measure. Because selective silencing of lipid handling genes including *lipoprotein lipase* (*LPL*) or *CD36* in ATM resulted in less LD formation in ATM as well as elevated circulating serum FFAs levels in mice fed with HFD [30]. On the contrary, enhancing the lipid storage capability of ATM by depletion of lysosome with chloroquine treatment decreased basal lipolysis and serum FFAs in obese mice [12]. In addition, HFD feeding to mice led to generation of a subset of metabolically beneficial ATM defined by markers of F4/80, CD11b, CD11c, and CD206 (termed as FBC ATM), featuring increased lipid uptake and catabolism mediated by the activation of phosphoinositide 3-kinase- (PI3K-) nuclear factor erythroid 2-related factor 2- (NRF2-) MARCO signaling pathway. Similarly, this lipid-buffering subset of ATM antagonized obesity-associated metabolic disorders to some extent in mouse models of obesity [18].

More importantly, activation of lipid handling signaling was vital for macrophages to accumulate in WAT. In a study of Sharma et al., myeloid-specific depletion of the neuroimmune guidance cue molecule netrin-1 in mice attenuated ATM accumulation in visceral WAT as well as obesity-associated metabolic dysfunctions, mainly due to improved lipid handling and metabolic function in residual ATM [31]. By genetic depletion of netrin-1 coding gene *Ntn 1* in a mouse model of obesity, adipose tissue inflammation and insulin resistance were reduced, due to restored migratory capacity and facilitated emigration of ATM [32]. Likewise, the group of Amit identified a subset of ATM with a special transcriptional signature of triggering receptor expressed on myeloid cell 2 (*TREM2*), lysosomal acid lipase (*LIPA*), *LPL*, *Cathepsin B* (*CTSB*), *Cathepsin L* (*CTSL*), fatty acid-binding protein (*FABP*)*4*, *FABP5*, lectin, galactoside-binding, soluble (*LGALS*)*1*, *LGALS3*, *CD9*, and *CD36* and characteristic of lipid metabolism and phagocytosis, which they named as “lipid-associated macrophages” (LAM) in human and mouse adipose tissue [33]. Deletion of *TREM2* abrogated the lipid uptake and storage function of LAM, as well as their recruitment to enlarged adipocytes for the assembly of CLS in obese mice, thereby enforcing weight gain, hypercholesterolemia, and glucose intolerance [33, 34]. TREM2 could sense a broad array of anionic and zwitterionic lipids to sustain the microglial response in Alzheimer's disease in mice [35]. However, the specific species and source of lipids sensed by ATM via TREM2 in obesity remained largely unexplored. Besides TG, unesterified free cholesterol was also deposited in adipocytes [36]. Cellular cholesterol efflux was initiated and propagated by ABCA1 and the ATP-binding cassette transporters G1 (ABCG1) whose expressions and functions might be rate-limiting for cholesterol efflux [37]. During obesity in mice, expression of ABCG1 was reported to be essential for M2-like macrophages to migrate towards CLS for handling the unesterified cholesterol released from dead adipocytes [38]. Additionally, succinate, a tricarboxylic acid (TCA) cycle intermediate which could serve as an activation signal in macrophages stimulated by lipopolysaccharide (LPS) [39], was overproduced from adipose tissue in mice under stressed conditions like hypoxia and hyperglycemia and induced upregulated chemotaxis in ATM towards CLS through activation of succinate receptor 1 (SUCNR1) [40].

In sum, we postulated that dysfunctional adipocytes would release a distinguished spectrum of lipid species or metabolites to signal ATM migration, retention, or proliferation in obesity, which might be another important mechanism underlining obesity-associated ATM accumulation. The effects of these lipid species or metabolites on ATM were possibly mediated by expressions of specific lipid sensors on cell surface of ATM. If so, blockage of these lipid sensors might be new therapeutic targets for obesity-related inflammation. Nevertheless, except results from genetically engineered mouse models, by far, there was no pharmacological component targeting these sensors in ATM. Therefore, further investigation is needed to characterize the spectrum of lipids or metabolites released from adipocytes during obesity, as well as to elucidate relevant signaling pathways in ATM.

### 2.3. Involvement of ATM-LD in Inflammation

Previous studies had demonstrated that inflammation of ATM compromised metabolic homeostasis in obese mice [3, 4, 21, 41]. ATM comprised proinflammatory M1 and anti-inflammatory M2 subsets. The M1-like ATM induced by T helper (Th) 1 mediators such as LPS were responsible for increased production of proinflammatory cytokines. However, the M2-like phenotype driven by Th2 mediators including IL-4 and IL-13 exhibited an anti-inflammation role with activation of the transcriptional factor peroxisome proliferator-activated receptor *γ* (PPAR*γ*) [41]. Obesity was associated with an increased ratio of M1/M2-like ATM in WAT, favoring generation of cytokines such as IL-6 and promoting adipocyte lipolysis, ectopic lipid accumulation, and insulin resistance [21].

A report employing transmission electron microscopy (TEM) showed that M1-like macrophages contained more LD than M2 phenotype [14], indicating lipid accumulation correlated with inflammation in ATM. In addition, within CLS, a novel subset of ATM defined by cell surface marker CD9 promoted inflammation after becoming lipid-laden in mice fed a HFD [42, 43]. In fact, inflammatory macrophages would increase the uptake of lipids [44]. In a study of Castoldi et al., cellular TG was demonstrated to be indispensible for macrophages' inflammatory role [45]. Moreover, elevation of VLDLR expression in ATM in obese mice increased the uptake of TG and C16:0 ceramides and rendered the polarization of ATM to M1-like phenotype, thus promoting adipose tissue inflammation and glucose intolerance [46]. These data suggested that cellular LD could be an important contributor to inflammation in ATM.

Then, how is ATM-LD linked with inflammation? Based on the current literature, the answer might be partly related to the metabolic reprogramming accompanied with the formation of LD in ATM. The result of Na et al. suggested that increased glycolytic capacity was required for the inflammatory phenotype of macrophages [47]. Specifically, the reliance of aerobic glycolysis by M1-like macrophages led to production of mitochondrial reactive oxidative species (ROS) and oxidative stress, which contributed to secretion of cytokines such as IL-6 and tumor necrotic factor-*α* (TNF-*α*) and adipose tissue inflammation [48–50]. Indeed, in patients with morbid obesity, disturbances in the antioxidant barrier and enhanced oxidative damage to proteins and lipids were observed. Although bariatric surgery improved redox homeostasis in obese patients without metabolic syndrome, those with metabolic syndrome showed a continuous decrease in the antioxidant status [51]. ROS accumulation would also lead to activation of Fgr kinase (a member from the Src family), which induced phosphorylation and activation of mitochondrial complex II and degradation of mitochondrial complex I, contributing to ATM polarization and obesity-associated insulin resistance in mice [52].

Moreover, LD formation was often associated with endoplasmic reticulum (ER) stress. Exposure of RWA264.7 to saturated fatty acid palmitic acid led to cytoplasmic accumulation of lipids, ER stress, and M1 polarization *in vitro* [53], while attenuation of ER stress reduced M1 polarization of ATM and subsequent metabolic stress in mice fed by HFD [53, 54]. On the contrary, in M2-like macrophages which were less lipid-laden, PPAR*γ* activation resulted in mitochondrial biogenesis and oxidative metabolism due to IL-4 stimulation and suppressed production of cytokines in mice [41, 55]. Interestingly, M2-like ATM were not using the LD-dependent TG hydrolysis pathway to generate FFAs for fatty acid oxidation. Instead, uptake of TG substrates via CD36 and subsequent lipolysis by LIPA was used to support elevated oxidative phosphorylation (OXPHOS), prolonged survival, and expressions of gene characteristic of M2 phenotype in mice [14]. What was more, promoting lipid metabolism via activation of PPAR*γ* and p62/Sqstm1 by palmitate partly limited inflammation in macrophages in adipose tissue, explaining why there was a low-grade sterile inflammation in obese mice [13]. However, van Dierendonck et al. reported lipid contents in ATM were significantly reduced in mice with myeloid-specific deficiency of hypoxia-inducible lipid droplet-associated (HILPDA). But in DIO in mice, this decreased lipid storage in HILPDA-deficient ATM did not alter inflammatory and metabolic parameters. Thus, the data presented by van Dierendonck et al. questioned the contribution of LD accumulation in ATM to obesity-associated inflammation and metabolic complications [56]. Xu et al. also reported that by depletion of lysosome, lipid contents increased in ATM in mouse perigonadal fat *in vitro*. But this lipid accumulation was not associated with a clear inflammatory induction in ATM [12].

Taken together, the role of cellular LD in ATM inflammation merits further validation. Possibly, the cellular LD per se do not lead to polarization of ATM, but the metabolic reprogramming shifting to aerobic oxidation under LD formation is crucial to ATM inflammation. A case in point was HILPDA deficiency which led to LD removal associated with increased oxidative metabolism in ATM [56]. It was also demonstrated that upregulation of oxidative metabolism in ATM by stimulation with recombinant growth differentiation factor 15 (GDF15) led to M2-like polarization, reversing adipose inflammation as well as insulin resistance in mice [57].

In light of the finding that adoptive transfer of bone marrow cells from VLDLR knockout mice into wild-type mice relieved adipose tissue inflammation and improved insulin resistance in DIO in mice due to reduced TG and ceramides uptake into ATM [46], certain subsets of ATM could be new pharmacological targets in obesity-related inflammation and metabolic complications, which warrants further investigation.

## 3. Reversal Remodeling of ATM during Lipolysis

### 3.1. ATM Accumulating and Becoming Lipid-Laden during Lipolysis

**Figure 2:**
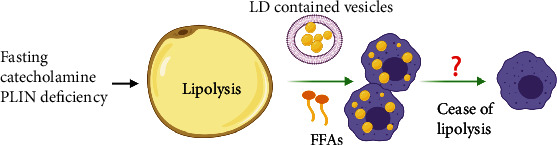
Scheme of transient ATM remodeling during lipolysis. Briefly, adipocyte lipolysis induced by fasting and catecholamine stimulation or PLIN deficiency leads to ATM accumulation and cellular LD abundance in ATM, possibly stimulated by LD contained vesicles and FFAs released from adipocytes. After lipolysis, remodeled ATM would return to normalization by unknown mechanisms.

Lipolysis induced by fasting resulted in mobilization of TG in adipocytes to maintain metabolism of other organs in the body [5]. Canonically, activation of *β*3-adrenergic receptor (ADRB3) in adipocytes by epinephrine or norepinephrine stimulated hydrolysis of TG to FFAs and glycerol mediated by cyclic adenosine monophosphate- (cAMP-) dependent protein kinase A (PKA) activation and subsequent phosphorylation of hormone sensitive lipase (HSL) [58]. In the process of lipolysis, ATM also underwent dramatic but transient remodeling, which showed certain similarities to that in the state of obesity (Figure 2).

On the one hand, accumulation of ATM in WAT was detected during lipolysis in normal lean mice. So what is the mechanism behind this alteration? Similar to obesity, lipolysis also resulted in the death of adipocytes and formation of CLS in normal mice [6, 15, 16]. Moreover, ATM accumulation was colocalized within CLS during lipolysis. Kim et al. reported increased formation of F4/80^+^ macrophage crown structure in gonadal WAT associated with augmented expression of *CD11B*, following activation of ADRB3 with isoproterenol treatment in mice [7]. While formation of CLS in obesity was known to persist for weeks or months, CLS induced by ADRB3 activation would quickly resolve and be updated by newly differentiated adipocytes during lipolysis *in vivo* in mice [15]. Thus, these data suggested that lipolysis-linked CLS formation might be one of the reasons responsible for the temporary recruitment of ATM in WAT. In a study by Lee et al., the result of triple staining of macrophage galactose-C type lectin 1 (MGL1, a marker of M2-like macrophages), LD enclosing protein PLIN1, and neutral lipid in whole-mount gonadal WAT showed that M2-like macrophages accumulated and surrounded LD in adipocytes devoid of PLIN1 in response to CL-316,243 treatment in mice, in order to function as a scavenger of dead adipocytes [15]. Thus, adipocyte death in CLS following ADRB3 activation could be a trigger for lipolysis-related ATM accumulation. Moreover, except the canonical manner, adipocytes could get rid of stored lipids directly by exocytosis of LD-contained vesicles during lipolysis in mice. These lipid-laden vesicles, after being internalized, would then induce migration, differentiation, and accumulation of ATM [59]. Unfortunately, the molecules and pathways involved were not well known by far.

On the other hand, metabolic reprogramming of ATM was also indicated by the results that lipolysis-derived FFAs or LD-containing vesicles eliciting neutral LD formation in ATM [5, 17, 59]. Functionally, some subsets of ATM were hypothesized to act as lipid buffer to prevent plasma TG from soaring up too much during fasting in mice [59]. In parallel to DIO, ADRB3 signaling could also lead to activation of the PI3K-NRF2-MARCO signaling pathway in myeloid, which contributed to accumulation of FBC ATM characteristic of facilitated lipid uptake and catabolism in mice [18]. Whether lipolysis could lead to activation of the lipid sensing signaling pathway in ATM similar to that in obesity as discussed above is worthy of further investigation. Of note, unlike in obesity where LD persisted in ATM, cellular lipid levels were restored in ATM post-lipolysis in mice, possibly due to ceasing of FFA flux. However, the specific mechanism is far from understanding. Since CLS were postulated to be an event initiating ATM remodeling in obesity, whether lipolysis-related temporary LD accumulation also associates CLS formation needs further validation. Meanwhile, it would be intriguing to answer the question how ATM were oriented to apoptotic adipocytes to form CLS in obesity or lipolysis. The discovery that the composition of FFAs generated by lipolysis in mice was similar to that released from dead adipocytes in cultured adipose tissue explant ex vivo [7] indicated the possibility that lipolysis might share with obesity a similar spectrum of adipocyte-secreted lipid metabolites in signaling ATM recruitment.

In sum, the available data suggested that lipolysis was associated with ATM remodeling characteristic of accumulation and metabolic reprogramming, possibly related to the role of ATM in clearance of lipid metabolites from adipocytes. Besides, the results also indicated the existence of mechanisms responsible for ATM renormalization postlipolysis which is not yet well investigated. In the following, we will address the alterations and implications of ATM during lipolysis in detail.

### 3.2. ATM Composition Altered by Lipolysis

Subpopulations of ATM were dynamically changed in lipolysis. As discussed above, deficiency in LD storage in adipocytes might trigger a signal for macrophages accumulation in lipolysis. Irregular and persistent lipolysis due to deficiency of PLIN1 in adipocytes initiated migration and M1-like polarization of monocytes into WAT in mice, mediated by lipid metabolites such as prostaglandin (PG) secreted from adipocytes. In this condition, macrophage clearance by clodronate alleviated inflammation in WAT in mice with PLIN1 deficiency [60]. Likewise, lipolysis in mice stimulated by CL-316,243 also led to PLIN1 deficiency in LD in adipocytes [15]. It was assumable that ADRB3 signaling resulted in ATM accumulation associated with M1 polarization. Intriguingly, during fasting in lean mice, Hu et al. reported that ATM accumulated but did not polarize, thus without altering the M1/M2 ratio of ATM in WAT. Nevertheless, it was demonstrated that adipocyte-derived prostaglandin E downstream of phospholipase A2 *α* (PLA2*α*) and cyclooxygenase 2 (COX2) activation played an important role in mediating ATM infiltration during adipocyte lipolysis ex vivo [61]. Allison et al. also demonstrated that recruitment of macrophages into fat depot was triggered by possible lipid metabolites derived from adipocytes in lipolysis both *ex vivo* and *in vivo*, followed by activation of the c-Jun N-terminal kinase (JNK)/nuclear factor kappa light chain enhancer of activated B cells (NF*κ*B)/COX2 axis [62]. In contrast, intermittent fasting which was associated with metabolic benefits was found to increase the population of M2 macrophages without affecting M1 population in mice fed with HFD [63]. Additionally, Asterholm et al. showed that 24 h fasting in healthy mice increased pan macrophages indicated by F4/80 expression in WAT, with M1-like macrophage decrease indicated by CD11c and nitric oxide synthase 2 (NOS2) expressions and M2-like macrophage increase indicated by CD206, CD301, and CD163 expressions. However, this dynamic switch to M2 phenotype during nutrition deprivation was impaired in obese mice [64]. Camell et al. also reported that the young fasted mice showed greater reduction in CD11c^+^CD206^−^ ATM (M1 population) with corresponding increase in CD11c^+^CD206^+^ ATM (M2 population) in comparison to fed ones [65]. Likewise, the team of James showed that chronic treatment of CL-316,243 for one week in mice increased M2 macrophages in gonadal WAT without affecting M1 macrophages [15]. The reason leading to these inconsistent data could be the composition of ATM in WAT was more complex than the M1/M2 diagram and distinct types of ATM dynamically adapted to different metabolic environments. Silva et al. found that a subset of ATM, termed as vasculature associated macrophages (VAM), was tightly associated with blood vessels in epididymal WAT. Remarkably, both fasting and ADRB3 activation could cause rapid depletion of VAM which would be restored by refeeding in healthy mice, confirming ATM subpopulations dynamically adapted to lipolysis-induced metabolic stress [66].

It is now clear that ATM composition in fat depots dynamically and transiently changes during acute metabolic stress such as fasting. But how exactly ATM subpopulations respond to lipolysis requires further characterization. Profound understanding of the mechanism responsible for ATM renormalization after lipolysis is of vital importance to prevent ATM accumulation in pathophysiological state-like obesity.

### 3.3. Role of ATM in Lipid Storage Efficiency of Adipocytes

The transient recruitment of ATM by lipolysis gives rise to a question concerning the significance of ATM in adipocyte lipolysis. According to the current literature, ATM could both positively and negatively influence lipid storage efficiency of adipocytes.

On the one hand, certain subsets of ATM were indispensible for maintaining lipid storage in adipocytes, as the demonstration that overall depletion of ATM by clodronate treatment promoted lipolysis and reduced fat weight in mice [5]. Thus, there might be a paracrine effect of ATM on lipid mobilization in adipocytes. Bu et al. revealed that CD11c^+^ ATM-derived growth differentiation factor 3 (GDF3), functioning as a ligand for activin receptor-like kinase 7 (ALK7) receptor on the surface of adipocytes, antagonized lipolysis via inhibition of lipase in mice [67]. In line with this result, age-associated impairment in fasting-induced lipolysis was partly accounted by activation of the Nod-like receptor (NLR) pathway and subsequent induction of GDF3 in ATM in visceral WAT in aged mice [65]. Moreover, M2-like ATM also had an inhibitory effect on lipolysis, with the impairment in their differentiation leading to lipolysis, diminished fat mass, and subsequent development of hypertriglyceridaemia as well as insulin resistance in mice, which could be restored by supplementation of M2-like ATM [68]. Nevertheless, the underlying mechanism responsible for actions of M2-like ATM on lipolysis remains elusive. Furthermore, certain subsets of ATM in CLS could induce quick differentiation of preadipocytes during lipolysis, since it was revealed that osteopontin (OPN) expressing M2-like ATM in CLS in mice attracted and initiated the differentiation of platelet-derived growth factor receptor alpha- (PDGFR*α*-) positive adipocyte progenitor that had a receptor CD44 for OPN [15]. Finally, the subpopulation of ATM expressing estrogen receptor *β* (ER*β*) improved lipid storage capability of adipocytes and had a suppressive effect on CLS formation via the axis of ER*β*/OPN/hypoxia inducible factor-1*α* (HIF-1*α*), with depletion of *ERβ* in ATM increasing the number of CLS in obese mice [69].

On the other hand, some subsets of ATM could also accelerate lipid depletion from adipocytes. It was well acknowledged that M1-like phenotype promoted lipolysis through secretion of cytokines like IL-6 and TNF-*α* [70]. Cytokines aside, ATM were able to secrete other molecules positively regulating lipolysis. Iwamura et al. reported that macrophage-derived soluble protein apoptosis inhibitor of macrophage (AIM) would induce ablation of PPAR*γ* activity in adipocytes in mice, leading to declined expressions of genes indispensable for LD coating and TG storage including fat-specific protein 27 (*FSP27*) and *PLIN* [71]. Moreover, activation of the chemokine (C-C motif) receptor 2-positive (CCR2^+^) macrophages was required for adipocyte death-triggered lipolysis in mice, associated with increased serum levels of catecholamine [7].

**Figure 3:**
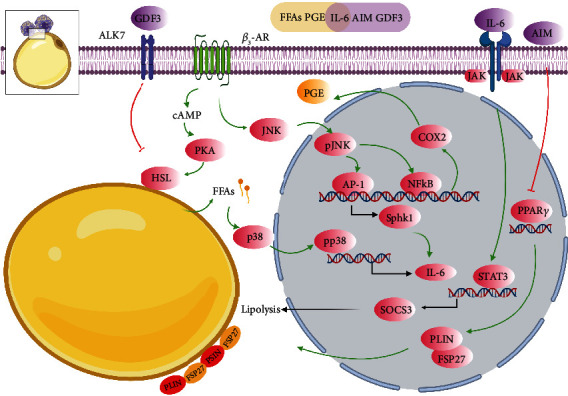
Scheme of relevant adipocyte alterations interacted with ATM in response to lipolysis. Representation of apoptotic adipocytes surrounded by ATM in CLS in lipolysis, with the small black square enlarging alterations of adipocytes. The signaling pathways that link these components by activation (green arrows) or inhibition (red inhibitory lines) are also indicated. Light yellow bar indicates secretome of adipocytes, while light purple one indicates secretome of ATM. Briefly, ADRB3 activation mediated by the canonical cAMP/PKA/HSL axis as well as the JNK/NF*κ*B/COX2 axis liberates various lipid metabolites like FFAs and PGE from adipocytes, which would trigger infiltration of lipolysis-associated ATM. In addition, ADRB3 signaling also leads to IL-6 production from adipocytes, through p38 activation stimulated by FFAs building up or downstream of the JNK/AP-1/SPHK1 signaling pathway. IL-6 could induce adipocyte remodeling by activation of the JAK/STAT3/SOCS3 signaling, further enhancing catabolism and liberation of lipids from adipocytes. Also, IL-6 is an important signal node for ATM accumulation. Bilaterally, ATM participate in affecting adipocyte lipolysis via secretion of different molecules. On the one hand, ATM-derived GDF3 can inhibit lipolysis via activation of ALK on cell surface of adipocytes and subsequent lipase inhibition. Additionally, AIM secreted from ATM leads to irregular and persistent lipolysis due to inhibition of PPAR*γ* and reduction of FSP27 and PLIN levels. On the other hand, M2-like ATM inhibit adipocyte lipolysis by undefined mechanisms.

It is likely that ATM of different subsets can secrete an array of molecules that have bilateral roles in the regulation of lipid storage efficiency of adipocytes. Therefore, further interrogation into the secretome of diverse subsets of ATM might be helpful for in-depth understanding of the crosstalk between adipocytes and ATM (Figure 3).

Compared to obesity, lipolysis is also associated with ATM remodeling in terms of composition and metabolism. Future experiments on the comparison and characterization of ATM remodeling in these two conditions will help to comprehend the persistence of ATM in obesity. Next, we decipher a potential molecule (IL-6), which showed homologous alterations in WAT in both obesity and lipolysis and might have a role in ATM remodeling.

## 4. Role of IL-6 in the Crosstalk between ATM and Adipocytes

As summarized above, dysfunctional adipocytes in obesity or lipolysis might drive chemotactic response of ATM via a secretome of lipids/metabolites which induced activation of lipid sensing pathways in ATM. If this assumption was true, upstream molecules involved in regulation of this process must exist. We focused on the role of adipose IL-6 in ATM remodeling in the context of obesity and lipolysis for the reasons as follows. First of all, both obesity and lipolysis induced a surge in IL-6 in adipose tissue in mice [16, 19, 20]. Secondly, both adipocytes and ATM can secrete IL-6 [72, 73]. The level of IL-6 in adipocytes and macrophages would be enhanced when coculturing these two cells ex vivo, which would become more prominent in the presence of TLR4 ligand LPS [74], suggesting a crosstalk between adipocytes and macrophages in IL-6 production [75]. Thirdly, emerging evidence supported that IL-6 was associated with ATM accumulation in obesity as well as lipolysis [76–78]. Finally, IL-6 performed an important role in regulating lipid homeostasis in adipocytes [79].

### 4.1. Effects of Adipose IL-6 on ATM in Obesity

IL-6 expression in adipose tissue was persistently elevated in obese mice [19]. In fact, obesity was demonstrated to be a positive regulator of IL-6 and IL-6 receptor levels in subcutaneous WAT, which contributed to induction of inflammation and obesity-associated metabolic dysfunctions in mice [80]. In addition, IL-6 *trans*-signaling through the soluble IL-6 receptor (sIL-6R) had a major proinflammatory effect, with circulating sIL-6R more closely associated with insulin resistance status than waist-to-hip ratio or body mass index (BMI) in morbidly obese Taiwanese adults [81]. This inflammatory role of IL-6 possibly involved ATM, as it was demonstrated that adipocyte IL-6 promoted local inflammation by increasing ATM accumulation as well as M1 polarization in WAT, with conditional depletion of *IL-6* in adipocytes ameliorating inflammation as well as glucose intolerance in mice fed a HFD [73]. On the contrary, blocking IL-6 *trans*-signaling with its inhibitor sgp 130Fc prevented HFD-induced ATM accumulation in mice [82]. Braune et al. reported that IL-6 was also produced by M2-like ATM in obese mice fed a HFD [83]. Paradoxically, IL-6/STAT3 signaling was required for M2-like ATM proliferation in obesity and could thus retard obesity-related insulin resistance in mice [84]. The augmentation and proliferation of M2 polarization by IL-6 were presumably due to upregulation of the IL-4 receptor *α* (IL-4 R*α*) expression via STAT3 activation. IL-6 signaling could act as a Th2 cytokine and antagonize inflammation via favoring proliferation of M2-penothype macrophages and consequent clearance of dead adipocytes in obese mice [83, 84]. Taken together, adipose IL-6 signaling would exert diverse functions depending on where it stemmed from, with adipocyte-derived IL-6 inducing ATM accumulation and inflammation in WAT, while IL-6 from M2-like ATM ameliorating inflammation by clearance of dead adipocytes.

So what is the ultimate effect of adipose IL-6 on inflammation in obesity? It might depend on the onset of obesity. At the early stage of obesity, IL-6 probably induces ATM polarization and inflammation, whereas it antagonizes inflammation at the late onset of obesity when CLS formations are dominant. However, the mechanisms responsible for elevated IL-6 in adipocytes or ATM under obesity remain largely unexplored and merit further investigation.

### 4.2. Effects of Adipocyte IL-6 on ATM in Fasting

Lipolytic activation also triggered a proinflammatory response in WAT, with acute activation of ADRB3 eliciting expressions of proinflammatory genes including *IL-6* in WAT in mice [16, 20]. Moreover, ADRB3 was demonstrated to be necessary and sufficient for acute stress-induced IL-6 production from brown adipocytes in mice [85]. In white adipocytes, building up of FFAs was proved to be a trigger for lipolytic signaling-induced IL-6 production through phosphorylation and activation of p38 downstream of HSL, with blockage of FFAs release minimizing the increased proinflammatory cytokines both *in vivo* and *in vitro* [86]. Furthermore, it was reported that expression of sphingosine kinase 1 (SPHK1) induced by activation of the JNK/activating protein-1 (AP-1) signaling pathway was indispensible for IL-6 production by ADRB3 in mice. Functionally, this IL-6 production was correlated with macrophage infiltration in epididymal fat during lipolysis in mice [76–78], which suggested a possible role of adipocyte IL-6 in ATM remodeling during lipolysis. Moreover, it was reported that lipolysis-derived FFAs per se only elicited formation of neutral LD but not inflammation in macrophages *in vitro* [17], supporting the possibility that other molecules like IL-6 released from adipocytes during lipolysis might trigger ATM recruitment and inflammation.

Therefore, lipolysis by ADRB3 activation could lead to IL-6 production in adipocytes partly mediated by the JNK/AP-1/SPHK1 signaling pathway (Figure 3). It would be fascinating to clarify whether obesity shares with lipolysis this mechanism to regulate IL-6 production in adipocytes.

### 4.3. Crosstalk between ATM and Adipocytes via IL-6

Except for its role in immunity, adipose IL-6 played a role in lipid homeostasis in both ATM and adipocytes. For one thing, IL-6 accelerated lipid removal from macrophages. IL-6 facilitated ABCA1-mediated cholesterol efflux from human macrophages to apolipoprotein AI (apoAI) via activation of the Janus kinase (JAK)-2/STAT3 signaling pathway *in vitro* [87]. The class A macrophage scavenger receptor (MSR) contributed to increased uptake of modified low density lipoproteins (LDL) and transformation of macrophages into lipid-laden foam cells. IL-6 dose-dependently inhibited the expression of MSR and reduced the uptake of acetylated LDL in THP-1 macrophages *in vitro* [88]. For another, IL-6 was also implicated in regulation of lipid metabolism in adipocytes [79]. In adipose tissue, IL-6 elevation could initiate a metabolic reprogramming. For example, in fasting state, metabolism was transiently switched from carbohydrate to lipid utilization in humans [89]. In this context, circulating IL-6 level was increased, contributing to TG mobilization and increased oxidation of FFAs in adipose tissue in mice [90]. In fact, exercise-induced IL-6 accelerated lipolysis and reduced visceral fat mass in humans [91]. Furthermore, IL-6 accounted for increased lipolysis in mesenteric adipose tissue in mice fed a HFD [21, 92]. Additionally, IL-6 was associated with cachexia-related lipolysis and WAT waste, with anti-IL-6 receptor antibody inhibiting WAT lipolysis in mice [93]. Mechanistically, IL-6 could induce the expressions of STAT3 and suppressor of cytokine signaling 3 (SOCS3) which inhibited insulin signaling in adipocytes [94]. Likewise, vesicle-contained IL-6 released from tumor would lead to activation of the JAK/STAT3 pathway and resulted in lipolysis in mice [95]. Hence, IL-6/STAT3 signaling had an important role in liberating lipids from adipocytes in various conditions. It was well known that ATM were major sources for increased systemic IL-6 levels in obesity in mice [49]. In fact, exposure to lipids or lipid metabolites such as PGE2 stimulated production of IL-6 in macrophages *in vitro* [96, 97]. As discussed above, a specific spectrum of lipid species or metabolites from adipocytes possibly functions as signals for remodeling and recruiting ATM during obesity or lipolysis. But the exact molecule signaling governing this process is scarcely known. Given the result that ATM surrounding dead adipocytes in CLS in obese mice expressed IL-6 [8], it is tempting to assume that ATM-derived IL-6 is one of the upstream molecules manipulating the secretion of those lipids/metabolites from adipocytes.

It is likely that there is a positive feedback loop between adipocytes and ATM via IL-6 signaling. Specifically, IL-6 stimulates lipid/metabolites release from adipocytes, which consequently recruits and remodels ATM into lipid-laden status. Then, ATM respond by releasing more IL-6 to get rid of their own lipids and further facilitate lipid liberation from adipocytes, thus forming a vicious circuit to enable ATM persistence. Rigorous investigation is needed to test this assumption.

## 5. Conclusions

ATM exhibited similar remodeling in terms of abundance and cellular LD contents in the contexts of obesity and lipolysis. Thus, comprehensive comparisons of ATM remodeling in these two situations might give rise to novel insight into obesity-associated persistence of ATM. Metabolic reprogramming associated with activation of lipid sensing signaling in ATM might be an important mechanism for ATM accumulation in obesity. Based on the comparison of ATM remodeling under obesity and WAT lipolysis, CLS formation seemed to be an overlapping event that initiated ATM remodeling, with a spectrum of lipids or metabolites released from dysfunctional adipocytes being potential signaling molecules. Thus, it is assumable that blockage of this lipid sensing signaling in ATM could antagonize obesity-related inflammation. ATM composition dynamically changed in response to metabolic stress signals from adipocytes, and characterization of distinct subpopulations of ATM is necessary for pharmacological targeting. How the transient abundance of ATM and their cellular LD contents are renormalized postlipolysis is poorly understood. There could be a positive feedback loop between ATM and adipocytes via IL-6 signaling which generates liberation of lipid metabolites from adipocytes to induce ATM remodeling, and ATM then in turn release more IL-6 enhancing its action on adipocytes. It should be noted that the majority of the reported information came from animal models. To verify these findings and develop new drugs, more data from human studies should be collected. Hopefully, investigation of adipose IL-6 signaling might be helpful in the discovery of the clearance mechanism of ATM from adipose tissue.

## Abbreviations

ABCA1: ATP-binding cassette transporter A1
ABCG1: ATP-binding cassette transporter G1
ADRB3: *β*3-adrenergic receptor
AIM: Apoptosis inhibitor of macrophage
ALK7: Activin receptor-like kinase 7
AP-1: Activating protein-1
apoAI: Apolipoprotein AI
ATM: Adipose tissue macrophages
BMI: Body mass index
cAMP: Cyclic adenosine monophosphate
CD36: Cluster of differentiation 36
CEC: Cholesterol efflux capacity
CLS: Crown-like structures
COX2: Cyclooxygenase 2
CTSB: Cathepsin B
CTSL: Cathepsin L
DIO: Diet-induced obesity
ER: Endoplasmic reticulum
ER*β*: Estrogen receptor *β*
FABP: Fatty acid-binding protein
FFAs: Free fatty acids
FSP27: Fat-specific protein 27
GDF3: Growth differentiation factor 3
GDF15: Growth differentiation factor 15
HFD: High fat diet
HIF-1*α*: Hypoxia inducible factor-1*α*
HILPDA: Hypoxia-inducible lipid droplet-associated
HSL: Hormone sensitive lipase
IL-6: Interlerkin-6
JAK: Janus kinase
JNK: c-Jun N-terminal kinase
LAM: Lipid-associated macrophages
LAMP: Lysosomal associated membrane protein
LD: Lipid droplets
LDL: Low-density lipoproteins
LGALS: Lectin, galactoside-binding, soluble
LIPA: Lysosomal acid lipase
LPL: Lipoprotein lipase
LPS: Lipopolysaccharide
MARCO: Macrophage receptor with collagenous structure
MCP-1: Monocyte chemoattractant protein-1
MGCs: Multinucleated giant cells
MGL1: Macrophage galactose-C type lectin 1
MSR: Macrophage scavenger receptor
MYD88: Myeloid differentiation primary response 88
NADPH: Nicotinamide adenine dinucleotide phosphate
NF*κ*B: Nuclear factor kappa light chain enhancer of activated B cells
NLR: Nod-like receptor
NOX2: NADPH oxidase 2
NOS2: Nitric oxide synthase 2
NRF2: Nuclear factor erythroid 2-related factor 2
OPN: Osteopontin
OXPHOS: Oxidative phosphorylation
PC: Phosphatidylcholine
PDGFR*α*: Platelet-derived growth factor receptor alpha
PG: Prostaglandin
PI3K: Phosphoinositide 3-kinase
PKA: Protein kinase A
PLA2*α*: Phospholipase A 2*α*
PLIN2: Perilipin 2
PPAR*γ*: Peroxisome proliferator-activated receptor *γ*
ROS: Reactive oxidative species
SOCS3: Suppressor of cytokine signaling 3
SPHK1: Sphingosine kinase 1
STAT3: Signal transducer and activator of transcription 3
SUCNR1: Succinate receptor 1
TCA: Tricarboxylic acid
TEM: Transmission electron microscopy
Th: T helper
TG: Triglyceride
TLR2: Toll-like receptor 2
TNF-*α*: Tumor necrotic factor-*α*
TREM2: Triggering receptor expressed on myeloid cell 2
WAT: White adipose tissue
VAM: Vasculature-associated macrophages
VLDLR: Very low-density lipoprotein receptor.
